# The pest kill rate of thirteen natural enemies as aggregate evaluation criterion of their biological control potential of *Tutaabsoluta*

**DOI:** 10.1038/s41598-021-90034-8

**Published:** 2021-05-24

**Authors:** Joop C. van Lenteren, Alberto Lanzoni, Lia Hemerik, Vanda H. P. Bueno, Johanna G. Bajonero Cuervo, Antonio Biondi, Giovanni Burgio, Francisco J. Calvo, Peter W. de Jong, Silvia N. López, M. Gabriela Luna, Flavio C. Montes, Eliana L. Nieves, Pascal Osa Aigbedion-Atalor, Maria B. Riquelme Virgala, Norma E. Sánchez, Alberto Urbaneja

**Affiliations:** 1 Laboratory of Entomology, Wageningen University, P.O. Box 16, 6700AA Wageningen, The Netherlands; 2 Department of Agricultural and Food Sciences, University of Bologna, Viale Fanin, 42, 40127 Bologna, Italy; 3 Biometris, Department of Mathematical and Statistical Methods, Wageningen University, P.O. Box 16, Wageningen, 6700 AA The Netherlands; 4 Laboratory of Biological Control, Department of Entomology, Federal University of Lavras, P. O. Box 3037, Lavras, MG 37200-000 Brazil; 5 Department of Entomology and Acarology, Luiz de Queiroz College of Agriculture (USP/ESALQ), Piracicaba, Brazil; 6 Department of Agriculture, Food and Environment, University of Catania, 95123 Catania, Italy; 7 Research and Development, Koppert Spain S.L. Calle Cobre, 22. Polígono Industrial, Ciudad del Transporte, 04745 La Mojonera, Almería, Spain; 8 Instituto Nacional de Tecnología Agropecuaria (INTA), Instituto de Microbiología y Zoología Agrícola (IMYZA), Ituzaingó, Buenos Aires, Argentina; 9 Centro de Estudios Parasitológicos y de Vectores (CEPAVE – CONICET, UNLP and CICPBA), Boulevard 120 e/ 60 y 64, 1900 La Plata, Argentina; 10 International Centre of Insect Physiology and Ecology (icipe), P.O. Box 30772-00100, Nairobi, Kenya; 11 Departamento de Tecnología, Universidad Nacional de Luján y Facultad de Agronomia,, Universidad de Buenos Aires, Rutas 5 y 7, 6700 Luján, Argentina; 12 Programa de Ecología Terrestre, Departamento de Ciencias Básicas e Instituto de Ecología y Desarrollo Sustentable (INEDES), Universidad Nacional de Luján, Rutas 5 y 7, 6700 Luján, Argentina; 13 Centro de Protección Vegetal y Biotecnología, Instituto Valenciano de Investigaciones Agrarias (IVIA), Carretera CV-315, Km 10’7, 46113 Moncada, Valencia, Spain

**Keywords:** Behavioural ecology, Population dynamics, Behavioural ecology, Population dynamics

## Abstract

Ecologists study how populations are regulated, while scientists studying biological pest control apply population regulation processes to reduce numbers of harmful organisms: an organism (a natural enemy) is used to reduce the population density of another organism (a pest). Finding an effective biological control agent among the tens to hundreds of natural enemies of a pest is a daunting task. Evaluation criteria help in a first selection to remove clearly ineffective or risky species from the list of candidates. Next, we propose to use an aggregate evaluation criterion, the pest kill rate, to compare the pest population reduction capacity of species not eliminated during the first selection. The pest kill rate is the average daily lifetime killing of the pest by the natural enemy under consideration. Pest kill rates of six species of predators and seven species of parasitoids of *Tuta absoluta* were calculated and compared. Several natural enemies had pest kill rates that were too low to be able to theoretically reduce the pest population below crop damaging densities. Other species showed a high pest reduction capacity and their potential for practical application can now be tested under commercial crop production conditions.

## Introduction

Since 1798^1^, one of the key questions in ecology has been how populations of animals and plants are regulated. From the 1930s to the 1990s several theories emerged, with as the two most important ones the density-dependent regulation of populations^2^ and population limitation by density-independent factors^3^. Heated debates took place at congresses and in the literature, which still continue. However, ecologists today are of the opinion that perfect density dependence does rarely or not occur, and that populations are regulated by interacting density-independent and density-dependent factors. For an extensive discussion of the population regulation debate see Turchin^4^. In several disciplines of applied ecology, population regulation theories are playing an important role, including nature conservation, restoration of biodiversity, ecology of reintroduction of locally extinct species to their previous habitats, invasive species biology and biological control. According to Bellows and Hassell^5^ “population equilibria and population regulation lie at the heart of biological control”. In biological control, an organism (a natural enemy) is used to reduce the population density of another organism (a pest)^6^, which contributes to manage native and exotic pests in natural and managed ecosystems. In this paper we discuss a new method for efficacy evaluation of candidate organisms for use in the reduction of populations of pests and apply this method to natural enemies of a serious, quickly spreading invasive pest over the world, the South American tomato moth *Tuta absoluta* (Meyrick) (Lepidoptera: Gelechiidae)^7,8^. Although the paper focuses on biological control of pests, the same method can be used, for example, to estimate population reduction effects of invasive organisms on native organisms in natural and agricultural ecosystems.

### The need for biological control of Tuta absoluta

The distribution of *T. absoluta* was limited to South America until 2006, then the species was accidentally introduced into Spain and currently occurs on most continents and is still spreading at a high rate^7,8^. Without control methods, the pest may lead to a complete loss of yield. As the larvae of the pest spend most of their time within the leaf or in the fruit, chemical control is difficult and demands up to five sprays per week and 36 times per production cycle of 12 weeks to be effective and this results in quick development of resistance to pesticides, environmental pollution and human health risks^9^. One of the alternatives for controlling *T. absoluta* is to use biological control, but finding an effective natural enemy for a new, invasive pest is not an easy task as a pest often has tens to hundreds of species attacking it^6^. *Tuta absolua* is said to be associated with almost 200 species of predators and parasitoids in their native and newly invaded areas^7,8^. Currently, only four species of all pest associated natural enemies are commercially used: the egg parasitoids *Trichogramma pretiosum* Riley (Hymenoptera: Trichogrammatidae) and *Trichogramma achaeae* (Nagaraja and Nagarkatti) are applied on a very small scale, and the predatory mirids *Nesidiocoris tenuis* Reuter (Hemiptera: Miridae) and *Macrolophus pygmaeus* (Rambur) are used on a larger scale^7,8^. All four species were found fortuitously (e.g. the mirid *N. tenuis* was found preying on *T. absoluta* in Spain by Urbaneja^10^ a few days after the first *T. absoluta* individuals were found in a tomato crop in 2009), or as a result of testing organisms that are easily mass produced (e.g. the egg parasitoid *T. pretiosum* in Latin America^11^). However, all four are not optimal for *T. absoluta* control, because release of the predator *N. tenuis* can cause serious plant damage^12^, *M. pygmaeus* needs alternative food to be able to reproduce^13^, and the parasitoids *T. pretiosum* and *T. achaeae* have to be released frequently and in very large numbers^14,15^. Therefore, other, more effective natural enemies are acutely needed, as well as efficient methods to identify promising candidates from the multitude of pest associated species. We propose a three step approach: (1) a quick scan procedure to separate clearly ineffective or hazardous species, from potentially promising candidates based on available information, (2) a phase during which the pest reduction capacity of the candidates selected in step 1 is determined, and (3) a final stage where the performance of the most promising candidates is tested under commercial tomato production conditions.

### Quick scan procedure to select promising candidates from the pool of pest associated species

We previously published a list of 15 evaluation criteria, ranging from issues as “climatic adaptation to area where natural enemy will be used” to “complexity of importation and registration procedures”, for a quick scan of all organisms said to be associated with *T. absoluta*^16^. Applying these criteria, 180 species may be removed from lists of potentially effective natural enemies of this pest mentioned in publications^8,16^. This is, among others, because either they (1) have not been shown to attack *T. absoluta*, (2) are very likely to cause unacceptable nontarget effects, (3) develop too slowly or not at all on the targeted prey, (4) are trapped by the glandular trichomes on tomato and die, (5) do not kill sufficient pest organisms, or (6) are too expensive to mass produce. An example related to the fourth cause is the predator *Orius insidiosus* (Say) (Hemiptera: Anthocoridae): although it may kill a considerable number of pest eggs, it is caught by the sticky hairs on the stems of tomato and dies within minutes after release on the plant. Too slow immature development, high immature mortality and very low predation rates on pest eggs by *Geocoris punctipes* (Say) (Hemiptera: Geocoridae) illustrate causes three and five. Slow immature development, high mortality and low parasitism rates also make the parasitoids *Dineulophus phthorimaeae* de Santis (Hymenoptera: Eulophidae) and, when not provided with alternative food, *T. pretiosum* unlikely successful candidates for control of the pest. The quick scan results in less than 20 species remaining on the list of potentially successful natural enemies^16^.

### The pest kill rate as an aggregate parameter to estimate pest reduction capacity

To obtain an idea of the biological control potential of the remaining 20 species, van Lenteren et al.^16^ proposed to rank them by the pest kill rate as aggregate evaluation criterion. The pest kill rate is the weighted daily average of the lifetime killing of the host or prey due to actions (predation, parasitism and nonreproductive prey and host killing) of a natural enemy. Tommasini et al.^17^ used a preliminary version of the pest kill rate to compare species of the genus *Orius* as predator of thrips pests. Next, van Lenteren^18^ explained that the kill rate could be used not only to rank the potential control capacity of predators, but also of parasitoids. Then, Biondi et al.^19^ calculated the host kill rate of the parasitoid *Bracon nigricans* Szépligeti (Hymenoptera: Braconidae), which included both parasitism and host killing due to stinging and host feeding. The pest kill rate, *k*_*m*_, they found (0.121) is much higher than the intrinsic rate of population increase, *r*_*m*_, of *B. nigricans* (0.052). Van Lenteren et al.^16^ explained why the *r*_*m*_, of predators is not a useful criterion for comparison of their biocontrol capacity: it only provides information on how quickly a predator population can grow and does not tell how many prey items it can kill. In parasitoids, the *r*_*m*_ can be used for comparison of species that do not kill hosts by nonreproductive activities. However, many species do not only eventually kill the host as a result of parasitism, but additionally kill hosts by feeding or stinging them for host examination^20,21^. Therefore, also in parasitoids it is better to use the pest kill rate for comparison of their pest reduction capacity.

### Use of the pest kill rate parameter to compare effectivity of predators and parasitoids of Tuta absoluta

During the past two years detailed life-table data for development, mortality, sex ratio, nymphal and adult predation, parasitism capacity, nonreproductive host killing and longevity for predators and parasitoids of *T. absoluta* have been collected by contacting researchers who might have these data and were willing to provide them for determination of pest kill rates. Such data are relatively rare as they need to include the above-mentioned details for each organism of the cohort that was studied, which is an expensive, time and space consuming effort. Sadly, these detailed data are often neither included in a publication, nor available in a data repository. Life-table data were obtained for six species of mirid predators, four species of parasitoids that only kill hosts by parasitism and three species of parasitoids that both kill hosts by parasitism and nonreproductive host killing (Table 1). These species would not all emerge as potential candidates for control of *T. absoluta* in classical and augmentative control programmes when evaluated with the criteria discussed in van Lenteren et al.^16^. However, by using the life-table data of these natural enemies it is possible to illustrate how the pest kill rate can be used in the future for comparison and ranking of the biological control capacity of predators and parasitoids.

**Table 1:** Natural enemies of *Tuta absoluta* used in this study.

Natural enemy	Short characterization	Reference / data
**Predators**		
*Campyloneuropsis infumatus* (Carvalho) (Hemip.: Miridae)	zoophytophagous^2^, polyphagous, preference for eggs and L1 of *T. absoluta* Neoptropical	van Lenteren et al. 2019 complete cohorts, low uncertainty^1^
*Engytatus varians* (Distant) (Hemip.: Miridae)	zoophytophagous, polyphagous, preference for eggs and L1 of *T. absoluta* Neotropical	van Lenteren et al. 2019 complete cohorts, low uncertainty
*Macrolophus basicornis* (Stäl) (Hemip.: Miridae)	zoophytophagous, polyphagous, preference for eggs and L1 of *T. absoluta* Neotropical	van Lenteren et al. 2019 complete cohorts, low uncertainty
*Macrolophus pygmaeus* (Rambur) (Hemip.: Miridae)	zoophytophagous, polyphagous, preference for eggs and L1 of *T. absoluta* Palearctic	Mollá et al. 2014; complete cohort immatures, adult predation incomplete, medium uncertainty
*Nesidiocoris tenuis* Reuter (Hemip.: Miridae)	zoophytophagous, polyphagous, preference for eggs and L1 of *T. absoluta* Paleotropic	Mollá et al. 2014; complete cohort immatures, adult predation incomplete, medium uncertainty
*Tupiocoris cucurbitaceus* (Spinola) (Hemip.: Miridae)	zoophytophagous, polyphagous, preference for eggs and L1 of *T. absoluta* Neotropical	Lopez et al. 2019; fertility life table complete, partial predation data, high uncertainty
**Parasitoids**		
*Bracon nigricans* Szépligeti (Hym.: Braconidae)	idiobiont^3^, synovigenic^3^, oligophagous, gregarious^7^ larval ectoparasitoid with non-reproductive host killing/host feeding, prefers L3-L4 of *T.absoluta;* Paleotropic	Biondi et al. 2013 complete cohorts, low uncertainty
*Dineulophus phthorimaeae* de Santis (Hym.: Eulophidae)	idiobiont, synovigenic, oligophagous, solitary^8^ larval ecoparasitoid with non-reproductive host killing/host feeding; prefers L3 of *T. absoluta*; America	Luna et al. 2010, Savino et al. 2012 complete cohort adults, incomplete immature data; medium uncertainty
*Dolichogenidea (Apanteles) gelechiidivoris* Marsh. (Hym.: Braconidae)	koinobiont^4^, proovigenic^5^, oligophagous, solitary larval endoparasitoid prefers L1 and L2 of *T.absoluta*; America	Aigbedion-Atalor et al. 2020 complete cohorts, low uncertainty
*Necremnus tutae* Ribes and Bernardo (= *N. artynes*) (Walker) (Hym.: Eulophidae)	idiobiont, synovigenic, oligophagous, predominantly solitary larval ectoparasitoid with non-reproductive host killing/host feeding; prefers L3 of *T. absoluta*; Palearctic	Calvo et al. 2013 complete cohorts, low uncertainty
*Pseudapanteles dingus* (Muesebeck) (Hym.: Braconidae)	koinobiont, moderately proovigenic, oligophagous solitary larval endoparasitoid America, main natural enemy spontaneously occurring parasitoid in tomato crops	Nieves et al. 2015 complete cohorts, low uncertainty
*Trichogramma pretiosum* Riley (Hym.: Trichogrammatidae)	proovigenic, polyphagous solitary endoparasitoid of eggs, concurrent non-destructive when host feeding food (honey) is available, America	Bajonero 2016 complete cohorts, low uncertainty
*Trichogramma pretiosum* Riley (Hym.: Trichogrammatidae)	proovigenic, polyphagous solitary endoparasitoid of eggs, concurrent non-destructive host feeding and non-reproductive host killing/feeding when no alternative food is available, America	Montes 2020 complete cohorts, low uncertainty
*Trichogrammatoidea bactrae* Nagaraja (Hym.: Trichogrammatidae)	proovigenic, polyphagous solitary endoparasitoid of eggs, Asia	Riquelme Virgala & Botto 2010 complete cohorts, low uncertainty

^1^uncertainty: low = all data needed to calculate pest kill rate available; medium = part of data needed to be estimated; high = many data needed to be estimated; complete cohort data were available for predation and fertility of the predators *C. infumatus*, *E. varians* and *M. basicornis*; for *M. pygmaeus* and *N. tenuis* complete cohort data were available for predation of the nymphal stages and fertility, and partial data for predation by adults; for *T. cucurbitaceus,* complete data were available for fertility and partial data for predation by nymphs and adults. For six of the seven species of parasitoids, complete cohort data were available for parasitism, non-reproductive host killing and fertility; for *D. phtorimaeae* complete cohort data were available for the adult stage, but some data for immature development had to be estimated; ^2^zoophytophagous = eats arthropods and feeds on plants; ^3^idiobiont = paralysis of host at oviposition, no further development of host;^4^koinobiont = host continues development after being parasitized; ^5^proovigenic = most eggs are mature at emergence; ^6^synovigenic = eggs mature after emergence; ^7^gregarious = more than one parasitoid can develop on a host; ^8^solitary = one parasitoid develops per host.

In this paper, pest kill rates of 13 predators and parasitoids of *T. absoluta* are calculated and compared. Moreover, species characteristics other than their pest kill rates, as well as information on their current commercial application is used to speculate which of the evaluated species might be promising candidates for biological control of this pest.

## Results

### Intrinsic rate of population increase, r_***m***_, of the pest, predators and parasitoids by the Birch and Lotka-Euler approaches

**Figure 1:**
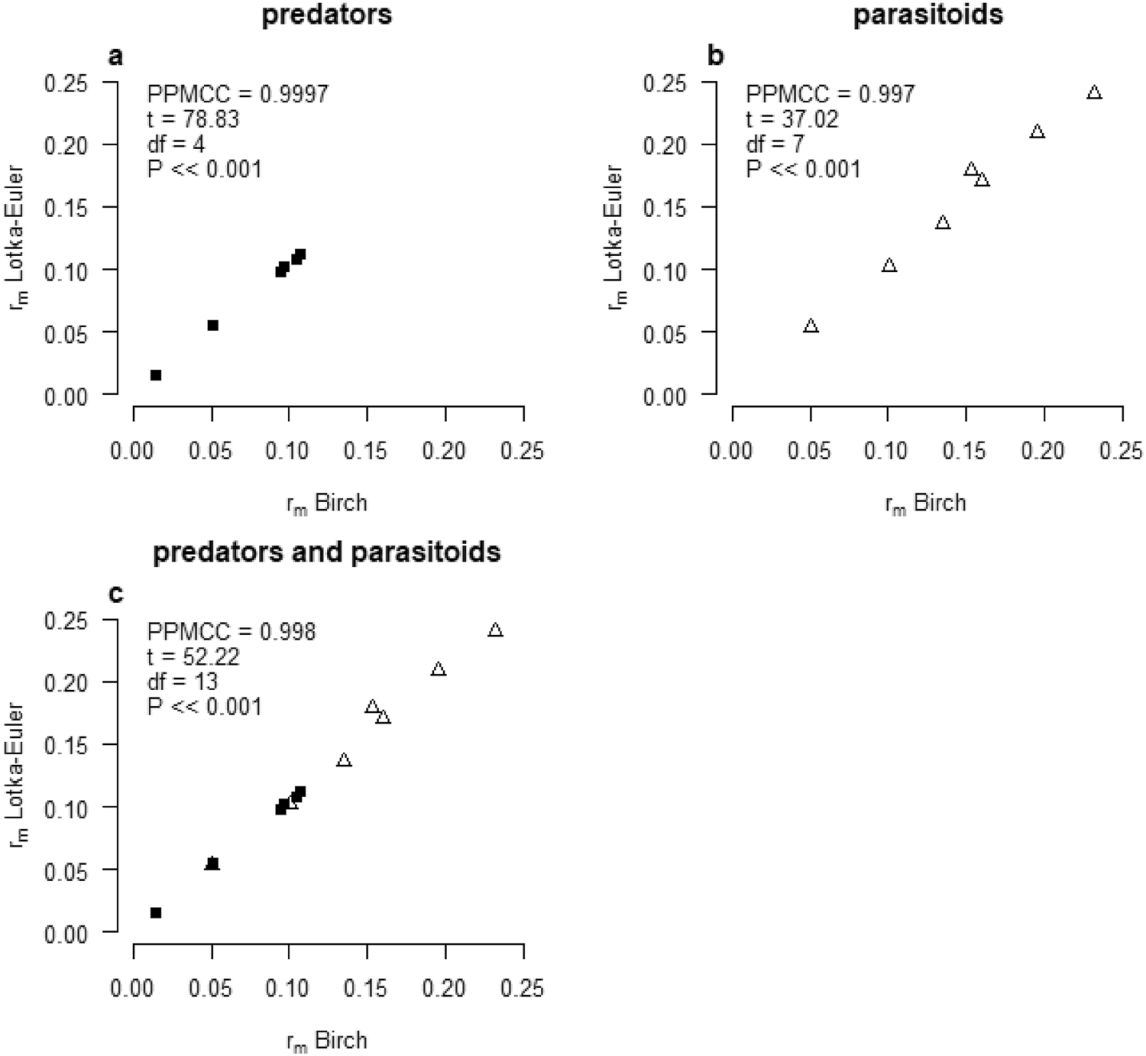
Correlation between values of *r*_*m*_ calculated by the Birch (x-axis) and the Lotka-Euler (y-axis) approach for predators (**a**), parasitoids (**b**) and predators + parasitoids (**c**).

Published values of the *r*_*m*_ of *T. absoluta* are summarized in supplementary material (Table S16), showing that most values are in the range of 0.13–0.19. For the natural enemies, the *r*_*m*_ calculated by the Lotka-Euler approach is higher than the *r*_*m*_ calculated by the Birch approach in all but one case (Table 2). The exception is *T. pretiosum* when offered no honey as food and this can be explained simply by the short life span of one day. Both for the predators and parasitoids, there is a strong and significant positive correlation between the *r*_*m*_ values calculated by both approaches (*P* <  < 0.001; Fig. 1). From now on, only the Lotka-Euler *r*_*m*_ values will be considered, because the Lotka-Euler approach results in a more precise estimate of *r*_*m*_ than the Birch approximation.

**Table 2:** Life-table parameters for six predator species and seven parasitoid species.

Species	*R*_*0*_	*T*	*r*_*m*_ Birch	*r*_*m*_ Lotka-Euler	*λ*
**Predators**					
*C. infumatus*	67.67	39.13	0.1077	0.1119	1.1183
*E. varians*	39.82	38.83	0.0949	0.0978	1.1028
*M. basicornis*	58.71	41.94	0.0971	0.1012	1.1065
*M. pygmaeus*	1.85	41.07	0.0149	0.0150	1.0151
*N. tenuis*	18.05	27.54	0.1050	0.1073	1.1133
*T. cucurbitaceus*	6.81	37.42	0.0513	0.0545	1.0560
**Parasitoids**					
B.nigricans*	4.44	29.73	0.0502	0.0542	1.0556
D.phthorimaeae*	0.67	22.40	− 0.0180	− 0.0181	0.9822
*D.gelechiidivoris*	16.45	27.78	0.1008	0.1021	1.1075
N. tutae*	32.00	22.69	0.1528	0.1785	1.1955
*P. dignus*	53.05	29.51	0.1346	0.1360	1.1457
*T. pretiosum*—food*	0.85	10.98	− 0.0151	− 0.0151	0.9850
*T. pretiosum*on on *Ephesitia* + food	23.01	16.06	0.1953	0.2085	1.2318
*T. pretiosum* F1 on *Tuta* + food	18.81	18.32	0.1602	0.1699	1.1851
*T. bactrae*	18.99	12.74	0.2311	0.2409	1.2746

*R*_*0*_ = net reproductive ratio; *T* = mean generation time; *r*_*m*_ = intrinsic rate of increase; *λ* = the finite rate of increase; * parasitoid with non-reproductive host killing/feeding; *r*_*m*_ values found for the pest *T. absoluta* are provided in supplementary material file S16, together with references for the papers presenting these values.

All the *r*_*m*_ values of the predators are lower than the *r*_*m*_ of the pest (Table 2 and Supplementary material Table S16) and, consequently, predator populations in the presence of ample *T. absoluta* eggs will be growing slower than the pest in the absence of natural enemies. The same reasoning holds for several parasitoid species. However, four out of the seven species of parasitoids have similar or higher *r*_*m*_ values than that of the pest: *N. tutae, P. dignus, T. pretiosum* with provision of honey as additional food, and *T. bactrae*. All natural enemies, except for *D. phthorimaea*e and *T. pretiosum* without additional food, have an *R*_0_ larger than 1 and a positive *r*_*m*_, so their population size will increase when only *T. absoluta* eggs and/or larvae are available for predation or parasitism. Tomato plants do not produce (extra-) floral nectar, but in a tomato crop other pests may be present that produce honey which can be used as additional food for *D. phthorimaea*e and *T. pretiosum*, resulting in higher *R*_0_ and *r*_*m*_, values. With the exception of *T. pretiosum* (Supplementary material Table S11), no experiments have been performed in tomato without additional food. The dramatic negative effect on the lifespan of and parasitism by *T. pretiosum* when honey is not offered as food is shown in Table 2.

### Pest kill rate, k_***m***_, of predators and parasitoids by the Birch and Lotka-Euler approaches

**Figure 2:**
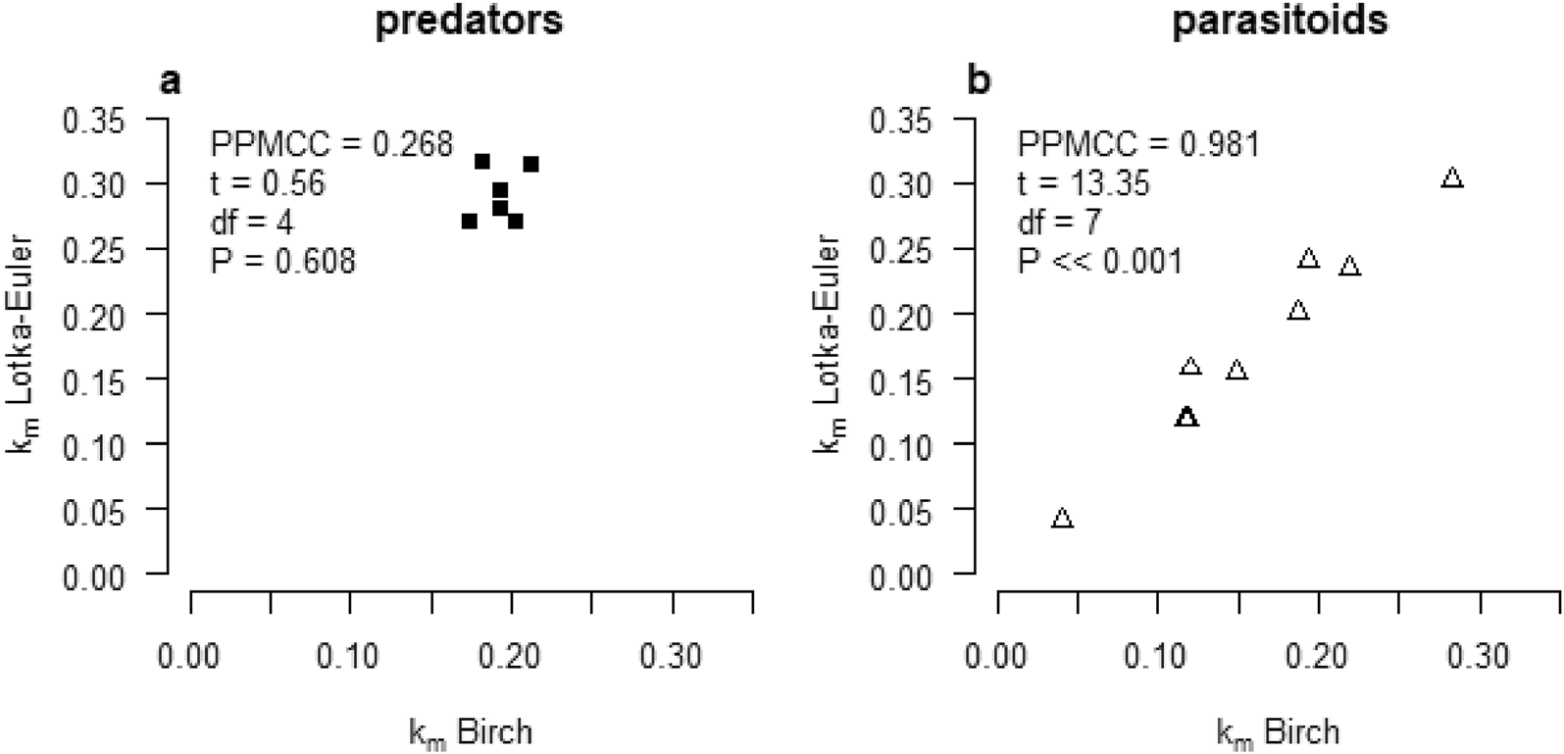
Correlation between values of *k*_*m*_ calculated by the Birch (x-axis) and the Lotka-Euler (y-axis) approach for predators (**a**) and parasitoids (**b**).

In all cases, the *k*_*m*_ calculated by the Lotka-Euler approach is higher than the *k*_*m*_ estimated by the Birch approach (Table 3), with the exception of *T. pretiosum* without food which is explained by the short life span of one day only. For the predators, there is a weak, nonsignificant, positive correlation between the *k*_*m*_ values calculated by both approaches (*P* = 0.608, Fig. 2), while the correlation is strong and positive for the parasitoids (*P* <  < 0.001, Fig. 2). From now on, only the Lotka-Euler *k*_*m*_ values will be considered, for the same reason as given in the section above.

**Table 3:** Life-table parameters related to the pest kill rate of predators and parasitoids of *Tuta absoluta.*

Species	*K*_*0*_	*T*_*k*_	*k*_*m*_ ln K_0_/T_k_	*k*_*m*_ Lotka-Euler
**Predators**				
*C. infumatus*	991.33	35.51	0.1942	0.2801
*E. varians*	748.04	31.34	0.1942	0.2940
*M. basicornis*	889.45	38.91	0.1745	0.2694
*M. pygmaeus*	445.06	30.14	0.2023	0.2708
*N. tenuis*	487.13	29.12	0.2125	0.3130
*T. cucurbitaceus*	543.90	34.57	0.1822	0.3152
**Parasitoids**				
B. nigricans*	29.89	28.16	0.1207	0.1578
D. phthorimaeae*	2.49	22.54	0.0405	0.0410
*D. gelechiidivoris*	25.95	27.78	0.1172	0.1189
N. tutae*	92.32	23.46	0.1929	0.2409
*P. dignus*	79.20	29.51	0.1482	0.1546
*T. pretiosum*—food*	3.64	10.95	0.1180	0.1180
*T. pretiosum* on *Ephestia* + food	33.84	16.06	0.2192	0.2355
*T. pretiosum* F1 on *Tuta* + food	30.64	18.32	0.1868	0.1998
*T. bactrae*	46.42	13.59	0.2823	0.3031

*K*_0_ = net consumption rate; *T*_*k*_ = mean predation/parasitization time; *k*_*m*_ = pest kill rate; * = species causing death of host by stinging and/or host feeding.

**Figure 3:**
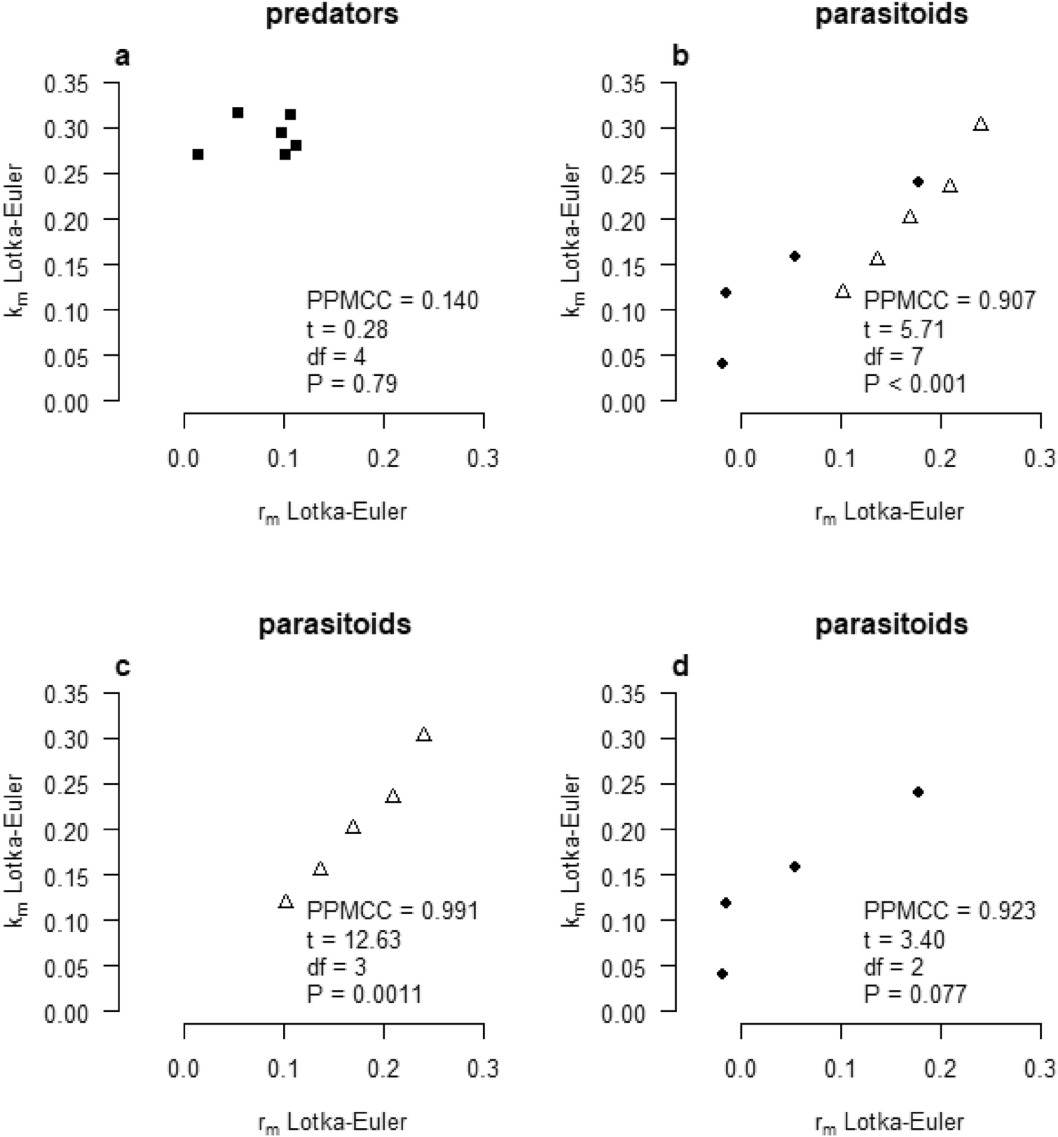
Correlation between *r*_*m*_ and *k*_*m*_ calculated by the Lotka-Euler approach for all predators (**a**), for all parasitoids (**b**), for parasitoids without nonreproductive host killing (**c**), and for parasitoids showing nonreproductive host killing (**d**).

The *r*_*m*_ and *k*_*m*_ values for predators show a weak, nonsignificant correlation (*P* = 0.79, Fig. 3a), while this correlation is strong for parasitoids (*P* <  < 0.001, Fig. 3b). The nonsignificant correlation found for predators can be explained by the fact that the total number of eggs killed has no linear relationship with the number of female offspring produced. This not only holds for *M. pygmaeus* and *T. cucurbitaceus* (high predation, very few offspring), but also for the other predators (high predation but varying numbers of offspring). Parasitoids that do not kill hosts by host feeding or stinging show, as expected, very small differences between *r*_*m*_ and *k*_*m*_ values (Fig. 3c), while parasitoids that do kill hosts by feeding and stinging show larger differences between *r*_*m*_ and *k*_*m*_ values, showing why it is important to calculate the pest kill rate *k*_*m*_ in order to estimate their total pest reduction capacity (Fig. 3d).

The *k*_*m*_ values of the six predators are all higher than the *r*_*m*_ of the pest (Table 3), which indicates that the predators may kill more eggs of *T. absoluta* than the latter can produce. The high kill rate, together with a considerable rate of population increase of the predators *C. infumatus*, *E. varians*, *M. basicornis* and *N. tenuis* is theoretically sufficient to reduce pest populations below the economic threshold density after a single or only a few releases. Two out of the seven species of parasitoids have similar *k*_*m*_ values as the *r*_*m*_ of the pest (Table 3): *P. dignus and T. pretiosum* (with provision of honey as additional food). The *k*_*m*_ values of the parasitoids *T. bactrae* and *N. tutae* are higher than the *r*_*m*_ value of the pest. This, together with these parasitoids’ high *r*_*m*_ values, suggests that they may be able to reduce pest populations below the economic threshold density after a single release.

**Figure 4:**
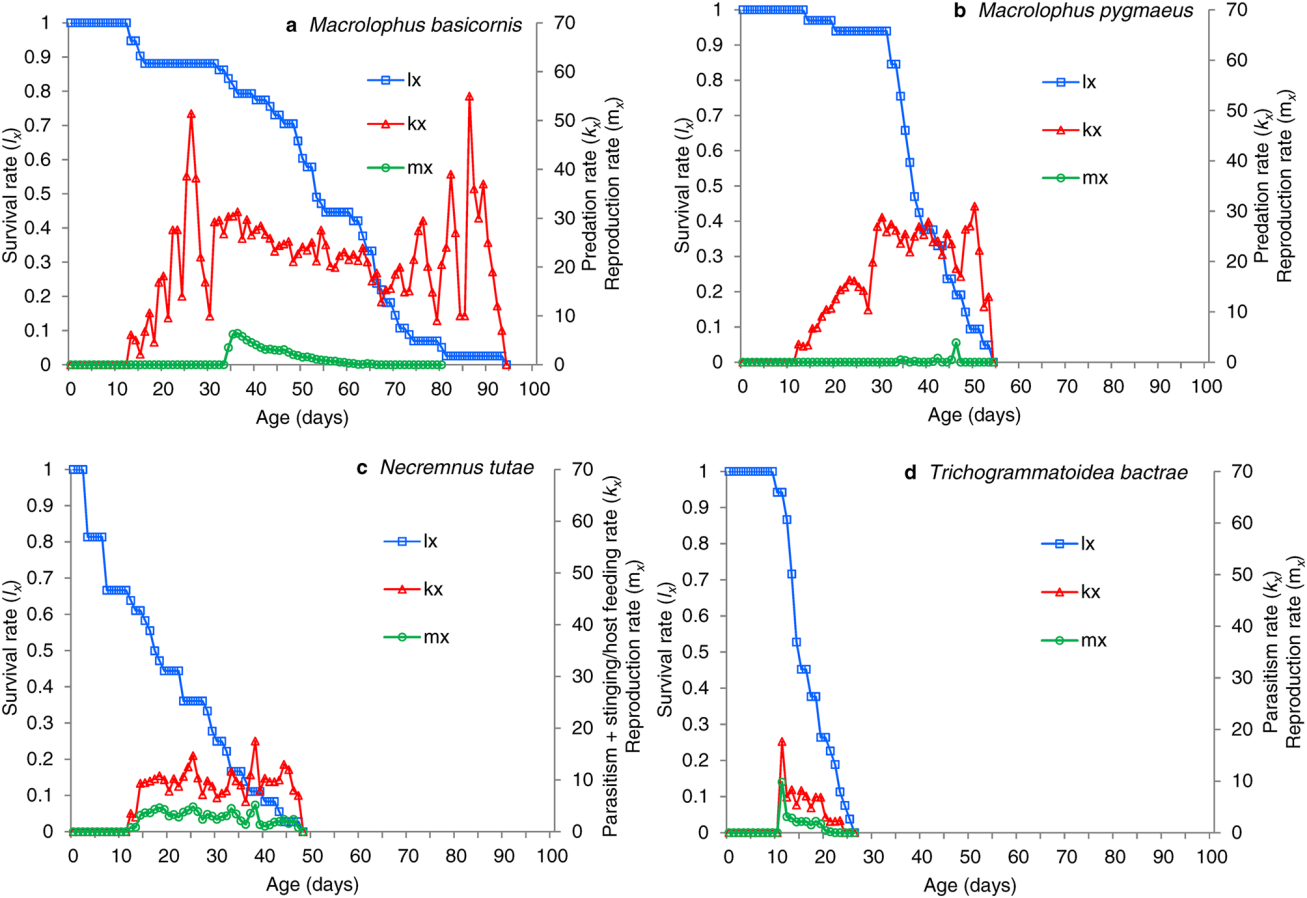
Examples of survival rate (*l*_*x*_), the reproduction rate (*m*_*x*_) and predation/parasitism rate (*k*_*x*_) values over the lifetime of selected species of predators and parasitoids of *Tuta absoluta.*

In Fig. 4, the survival rates (*l*_*x*_), the reproduction rates (*m*_*x*_) and the killing activity (*k*_*x*_) values over the lifetime of several *T. absoluta* natural enemies are presented. The supplementary material provides these figures for each species of predator and parasitoid. *Macrolophus basicornis* (Fig. 4a) is an example of a predator that lives, reproduces and preys during a long period of time; *M. pygmaeus* (Fig. 4b), is an example of a predator that lives, reproduces and preys during a short period of time. This translates to a very low *r*_*m*_ for *M. pygmaeus*, but the *k*_*m*_ is similar to that of *M. basicornis* because the predation rate is high during the short adult lifespan of *M. pygmaeus*. *Necremnus tutae* (Fig. 4c), is an example of a larval parasitoid that kills hosts through parasitism and by nonreproductive activities like stinging and host feeding. This parasitoid has a long adult life, and high *r*_*m*_ and *k*_*m*_ values. *Trichogrammatoidea bactrae* (Fig. 4d), is an example of an egg parasitoid that only parasitizes eggs, has a short adult life, but exhibits the highest *r*_*m*_ and *k*_*m*_ values of all parasitoids. The life tables in the supplementary material (Tables S1–S14) and the examples in Fig. 4 also show that predators, with the exception of *M. pygmaeus*, live longer (averages from about 50 to more than 90 days) and kill pest eggs over longer periods (averages from 40–80 days) than parasitoids (average lifespans from 11 to about 60 days, average parasitism/host kill periods from 1 to 15 days; with *B. nigricans* as the exception with a 64 days long maximal lifespan and a pest killing period of more than 50 days). Immature development times of predator and parasitoid species range from 10 to 28 days, and the average duration of a generation is longer for predators (from 40 to 60 days) than for parasitoids (from 11 to 37 days).

## Differences between Birch and Lotka-Euler approaches

The Birch approximation is easier to apply than the Lotka-Euler iteration, but it results in an underestimate of both the *r*_*m*_ and *k*_*m*_ due to the approximation of *T* and *T*_*k*_. The approximation results in higher values for *T*_*k*_ than for *T*, particularly in predators, because predation is spread over a much longer time (all nymphal stages and the adult stage) than reproduction. This translates to a higher underestimate for *k*_*m*_ than for *r*_*m*_ and to the highest underestimate in absolute terms for *k*_*m*_ in predators. In parasitoids, the difference between the two values for *r*_*m*_ and *k*_*m*_ calculated by both approaches is the smallest for those species that only kill hosts as a result of parasitism. The differences are larger for parasitoid species that do kill hosts by nonreproductive activities because this host killing is additional to killing by parasitism and takes place over a longer period than the parasitization activities. This leads to the choice to use *r*_*m*_ and *k*_*m*_ values calculated by the Lotka-Euler approach, and is discussed in more detail in van Lenteren et al.^16^. Another illustration that the Lotka-Euler iteration results in better estimates of the pest kill rate is the weak correlation between the differently estimated *k*_*m*_ values for predators (Fig. 2a). This is because in the Birch approach the *T*_*k*_ is calculated as an average estimate concentrated at a single point in time, while predation occurs over a long period and during both the nymphal and adult stages.

## Discussion and conclusions

The question of what would be the most effective natural enemy of *T. absoluta* is not easy to answer, as tomato production conditions and the pest spectra on tomato are widely varying across the world. A multitude of factors, including multitrophic and intraguild interactions, availability of alternative prey/hosts/sugar sources, host plant resistance, irrigation, fertilization, neighbouring crops^7,22,23^ will influence the control effect of a natural enemy. However, if only the pest kill rate is considered, the predators *N. tenuis* and *T. cucurbitaceus* would rank as potentially most effective, but all predators have a *k*_*m*_ higher than the *r*_*m*_ of the pest. Successful control of *T. absoluta* has been confirmed in the case of *N. tenuis*^15,24^. In addition, *C. infumatus* and *M. basicornis* successfully established on *T. absoluta* infested tomato plants and significantly reduced pest numbers^25^. The low *r*_*m*_ of *T. cucurbitaceus* and the very low *r*_*m*_ of *M. pygmaeus* indicate that these predators may only be used in inundative release programmes or play a role in natural and conservation biological control. *Trichogrammatoidea bactrae* would rank as the potentially most effective parasitoid, with *N. tutae*, *P. dignus*, and *T. pretiosum* showing kill rates similar to or higher than the population growth rate of the pest. The control capacity of *N. tutae* is confirmed by results published by Calvo et al.^26^ who observed that *N. tutae* significantly reduced *T. absoluta* populations, although relatively high release rates were needed. Interestingly, *N. tutae* often spontaneously invade greenhouses and successfully controlled *T. absoluta* in Spain^27^. The other parasitoids discussed in this paper could play a role in inundative release programmes or in natural and conservation biological control. The least effective natural enemy would be *D. phthorimaeae* with a *k*_*m*_ far below the *r*_*m*_ of the pest. We are now preparing a population dynamics model (to be presented elsewhere) to estimate the effect of the kill rate of the predator together with its population growth rate to be able to draw more precise conclusions about the potential role of the natural enemies in controlling *T. absoluta*. Also, not only the pest kill rate in combinatin with the population growth rate should be considered when answering the question about the best and worst natural enemy and this is discussed below.

As explained in the introduction, the main aim of using the pest kill rate parameter is to rank natural enemies after they have first been evaluated by the list of criteria given in van Lenteren et al.^16^. When these criteria are applied to the species discussed in this paper for use in seasonal inoculative (one or few releases when pest is observed or not yet present) or inundative (frequent releases of large numbers) programmes in a tomato crop with only *T. absoluta* as pest, *B. nigricans*, *D. phthorimaeae*, *D. gelechiidivoris* and *T. pretiosum* without provision of additional food would not be considered candidates for its control. Their population development is too slow and their capacity to parasitize and kill hosts too limited. The following species would be considered problematic as well: *M. pygmaeus* because it hardly reproduces on *T. absoluta*^13^ and *N. tenuis* because of its severe plant damaging effect due to phytophagy if its population density becomes too high^12^. The following candidates offer potential: the predators *C. infumatus*, *E. varians*, *M. basicornis* and *T. cucurbitaceus,* and the parasitoids *P. dignus*, *N. tutae*, *T. pretiosum* and *T. bactrae.* However, all parasitoids, with the exception of *T. pretiosum* without food, have been tested with provision of honey. It is not yet known how they would perform in a tomato crop with only *T. absoluta* as pest, where they have no access to nectar or honeydew. On the other hand, it is rather unrealistic to consider a situation with only *T. absoluta* as pest. In greenhouses in temperate climates occurrence of other pests such as whiteflies, aphids, lepidopterans and dipteran leafminers, thrips and spider mites is common^28–30^. As a result, honeydew is usually present, as well as alternative prey for the predators (e.g. see Fig. 2 in Biondi et al.^7^). In (sub)tropical greenhouses and in open field tomato production an even larger pest spectrum occurs^22,23,31^. If alternative prey is available, *M. pygmaeus* might still play a role as they kill large numbers of *T. absoluta* eggs and can reproduce on other prey. When honeydew is available the parasitoids *T. pretiosum* and *T. bactrae* might also be considered as candidates. This, then, results in all six species of predators (provided that *N. tenuis* populations are properly managed) and four species of parasitoids (*N.tutae*, *P. dignus*, *T. pretiosum* and *T. bactrae*) as candidates for seasonal inoculative or inundative release for control of the pest. Some of these species, when environmental risk assessments do not show serious nontarget effects, can be used also in classical biological control. The parasitoids that develop too slowly and/or kill too few hosts (*B. nigricans*, *D. phthorimaeae* and *D. gelechiidivoris*) might play a supportive role in conservation and classical biological control.

Part of the selection process for a natural enemy to be useful in commercial releases is its ease of mass rearing and whether the natural enemy is able to control other pests. Concerning the first issue, ease of mass rearing , economic mass-production methods have been developed earlier by commercial companies for mirid predators. The same holds for the parasitoids *T. pretiosum* and *T. bactrae*, but *N. tutae* is very difficult to mass rear^32^. The second issue, being able to control other pests, depends on the complexity of the pest situation in greenhouses. The pest spectrum in temperate greenhouses is much narrower than in the (sub)tropics, so both generalist predators or the more specific parasitoids could be used. In the (sub)tropics generalist mirid predators might be of greater value, as they not only kill a wide range of lepidopterans, but also whiteflies, aphids and even spider mites (V.H.P. Bueno, F.C. Montes and D. B. Silva, personal communication 2021,^33^). Bueno (personal communication 2020, and supplementary material (Table S17)) concluded that more than 15 different species of natural enemies would be needed for the control of more than 15 species of pests that occur on tomato in Brazil. With an effective Neotropical mirid predator, the number of species of natural enemies needed in tomato in Brazil might be reduced to less than five. A. Urbaneja (personal communication 2021) confirms that the same reasoning holds for the Paleotropic mirid *N. tenuis* with regard to control of a similar range of pests on tomato in the Mediterranean Basin.

An interesting characteristic of mirid predators besides their broad prey menu is that they are champions in dealing with the poisonous sticky hairs on tomato^34,35^. Yet another attractive characteristic is their capacity to induce defensive plant responses due to their phytophagous behaviour^36^. These defenses can activate the production of secondary metabolites and proteins that have toxic, repellent, and/or antifeedant effects on herbivores (direct defenses). Furthermore, the production and release of Herbivore-Induced Plant Volatiles can modify the behaviour of both phytophagous pests and their natural enemies (indirect defenses). Mirid-induced defenses may reduce the impact of pest herbivores on tomato plants, e.g. those induced by the *N. tenuis* might partly explain the success achieved by this predator in southeastern Mediterranean tomatoes^36^.

Eventually, only well-replicated experiments and/or well-documented experience under practical tomato growing situations will provide an answer to the suitability of these natural enemies for control of *T. absoluta*.

## Concluding remarks

1. As pest kill rate (*k*_*m*_) values are always higher than intrinsic rate of natural increase (*r*_*m*_) values, they provide better insight for estimating the potential of a natural enemy to control the pest. With the pest kill rates calculated in this paper, several species of natural enemies can be removed from the list of potential candidates for biological control of *T. absoluta* in seasonal inoculative or inundative release programmes. The few natural enemies that have shown to be able to control *T. absoluta* on tomato, in particular the predator *N. tenuis* and the parasitoid *N. tutae*, have *k*_*m*_ values that are considerably larger than the *r*_*m*_ of the pest.
2. To evaluate the potential of natural enemies in controlling the pest during several generations, a model linking the intrinsic rate of natural increase and the pest kill rate needs to be developed. With this model, it would be possible to estimate whether a seasonal inoculative or an inundative biological control programme is required.
3. Authors stating that a certain organism might be a promising candidate for biological control should support such statements with a critical evaluation of natural enemy characteristics, provision of quantitative data such as the pest kill rate as presented in this paper, and with results of experiments performed under realistic crop production conditions to confirm laboratory findings. When using evaluation criteria and the pest kill rate parameter, it is, for example, possible to eliminate more than 180 of the 200 species that are currently listed as candidates for control of *T. absoluta*. Over the years, many authors speculated about the contribution to biological control by the organism they studied without presenting data that make it possible to substantiate and confirm their supposition. Other authors mentioning *r*_*m*_—and in a few cases *k*_*m*_—values did not present the raw data as supplementary material or in a data repository, were not willing to provide the data upon request or, in some cases, the provided data indicated that the paper contained mistakes and that these raw data proved unsuitable to calculate the pest kill rate because they were incomplete. However, most requests for sending us detailed life-table data resulted in provision of these raw data. Thus, we petition for compulsory provision of raw data with the submission of a manuscript.

## Material and methods

### Predators of *Tuta absoluta*

In South America, more than 50 species of predators have been found in association with *T. absoluta* but less than 10 species might be sufficiently effective for its control^8,24,25^. Currently, the mirid *Tupiocoris cucurbitaceus* (Spinola) (Hemiptera: Miridae) is used for control of *T. absoluta* in Chile^37^ and for control of whitefly in Argentina and Uruguay (Carlos Silvestre, Brometan Biological System—Biobest Argentina, personal communication). Complete sets of life-table data are rare for predators, because the nymphal and adult stages consume many *T. absoluta* eggs per day (up to on average 70 eggs) and in their total lifetime (up to on average 1265 eggs). Comprehensive life tables have been published for three Neotropical mirids (*Campyloneuropsis infumatus* (Carvalho), *Engyttatus varians* (Distant) and *Macrolphus basicornis* (Stäl) (Hemiptera: Miridae))^16^, and sufficient predation data are available for the mirid *T. cucurbitaceus* to be able to reconstruct a life table^38^. Ten arthropod species, mainly hemipterans are known to prey on *T. absoluta* in Europe^8^. For two of these European mirid predators, *M. pygmaeus* and *N. tenuis*, detailed life-table data are available for predation by the nymphal stages^13^, and adult predation could be estimated based on partial daily predation data determined by Molla et al.^13^ and data earlier published^10,39–43^. A recent paper^44^ showed that the predation rates of nymphs and adults of the damsel bug *Nabis pseudoferus* Remane (Hemiptera: Nabidae) are similar to those of mirid predators, but the paper contained insufficient information to be able to calculate the pest kill rate.

### Parasitoids of *Tuta absoluta*

More than 50 (morpho) species of egg, larval and pupal parasitoids are associated with *T. absoluta* in South America, but only 23 could be confirmed parasitizing this host^45^. Almost 50 species of parasitoids are supposed to parasitize *T. absoluta* in Europe^8^. Recently, stocktaking of parasitoids in Africa^46^ resulted in finding several new species. Of all these parasitoids, few species have been tested under (semi-)practical conditions: the egg parasitoids *T. achaeae *in France and Spain^8^, *T. pretiosum* in Brazil^11^, and the larval parasitoid *Necremnus tutae* (= *N. artynes*) Ribes and Bernardo (Hymenoptera: Eulophidae) in Spain^26^. Only *Trichogramma* and *Trichogrammatoidea* egg parasitoids are mass reared and augmentatively released on a small area in the southwestern Mediterranean basin^7,8^ and in Brazil, Chile, Colombia, Ecuador and Peru in Latin America^45,47^. Detailed life-table data could be obtained for *T. pretiosum* with^14^ and without food (Montes, personal communication 2020, supplementary material Table S11). A third set of complete life-table data exists for *T. pretiosum*^48,49^, as well as a set for *T. achaeae* (T. Cabello, University of Almeria, personal communication 2018), but the original data sets were not made available. A complete life table was provided for *Trichogrammatoidea bactrae* Nagaraja (Hymenoptera: Trichogrammatidae)^50^, an exotic species initially introduced into Argentina for control of *Pectinophora gossypiella* Saunders (Lepidoptera: Gelechiidae)^45,50^.

In South America, most studies on larval parasitoids of *T. absoluta* concern *D. phthorimaea*, *Dolichogenidea* (*Apanteles*) *gelechiidivoris Marsh*. (Hymenoptera: Braconidae) and *Pseudapanteles dignus* (Muesebeck) (Hymenoptera: Braconidae)^45^. In Europe, several larval parasitoids have been studied, including *B. nigricans* and *N. tutae*^8^. Recent studies in Africa concentrated on *D. gelechiidivoris*, which was imported from Peru into Kenya^51^, and on *B. nigricans* and *Dolichogenidea appellator* (Telenga) (Hymenoptera: Braconidae)^46^. The host kill rate of the *Neochrysocharis formosa* (Westwood) (Hymenoptera: Eulophidae) was recently published^52^, but important basic information to judge the quality of the data was lacking in the paper and contact with the authors did not result in the provision of the raw data used for calculation of the kill rate by us. Complete sets of life-table data could be obtained for the larval parasitoids *B. nigricans*^19^, *D. gelechiidivoris*^51^, *N. tutae*^53^, and *P. dignus*^54^. Partial life-table data for *D. phthorimaeae*^55,56^ combined with additional information provided by these authors, resulted in sufficient material to determine the pest kill rate for this species.

In total, we obtained life tables for 13 species of natural enemies: six predators and seven parasitoids (Table 1). All cohort-based life-table data for each species together with a reference to the original paper details on material and methods are provided as supplementary material (supplementary material Tables S1–S14). Only life-table data have been used that were determined at temperatures between 24 and 26 °C. Although the experimental conditions differed with regard to humidity, photoperiod, light intensity, ventilation and size of the experimental arenas, we do not expect they strongly influenced the life-table data which is explained in supplementary material Text S15.

### Research involving plants

The plants used in the experiments were commercially available cultivars and did not involve plant species at risk of extinction or species of the wild flora. Our research complied with local and national regulations—Formal ethical approval was not required.

## Calculation of intrinsic rate of population increase (***r***_***m***_) and pest kill rate (***k***_***m***_)

Life-table parameters of predators and parasitoids were studied following the methodology explained by Birch^57^, where *x* denotes the pivotal age in days after an egg is laid (so, including immature stages for predators), *l*_*x*_ the age-specific survival (including immature mortality), and *m*_*x*_ the age-specific fertility (the number of females produced per female alive at age *x*). These were determined in order to calculate the derived quantities as defined in Table 4. The pest kill rate (*k*_*m*_) of the predators and parasitoids was calculated using the same formula as *r*_*m*_, but the age-specific fertility (*m*_*x*_) was substituted with the age-specific predation (*k*_*x*_) both during the nymphal and the adult stages for predators and only the adult stage for parasitoids. The mean predation time *T*_*k*_ can be considered as the time required for a population to predate at a rate of *K*_*0*_. The net consumption rate *K*_*0*_ is the number of prey items killed during a generation of the predator, corrected by natural mortality of the predator. For parasitoids, *m*_*x*_ was substituted by the age-specific parasitism, and, if parasitoids showed nonreproductive host killing due to stinging and/or host feeding, by the age-specific host killing. *K*_*0*_ for parasitoids is the number of hosts killed by parasitism and nonreproductive host killing, corrected by natural mortality of the parasitoid.

**Table 4:** Description and formulas for the data-based quantities.

Notation	Description
*x*	Pivotal age in days after an egg is laid
*l*_*x*_	Daily survival at age *x* (including immature mortality)
*m*_*x*_	Daily fertility (the number of females produced per female alive at age *x*)
$R_{0} = \mathop \sum \limits_{x = 0}^{{x_{max} }} l_{x} m_{x}$	Net reproductive ratio
$T = \frac{{\mathop \sum \nolimits_{x = 0}^{{x_{max} }} l_{x} m_{x} x}}{{R_{0} }}$	Mean generation time
$r_{m} = \frac{{{\text{ln}}\left( {R_{0} } \right)}}{T}$	Intrinsic rate of population increase
$\lambda= {\text{exp}}\left( {r_{m} } \right)$	Finite rate of increase
*k*_*x*_	Daily predation/parasitim/nonreproductive host killing at age *x*
$K_{0} = \mathop \sum \limits_{x = 0}^{{x_{max} }} l_{x} k_{x}$	Net consumption rate
$T_{k} = \frac{{\mathop \sum \nolimits_{x = 0}^{{x_{max} }} l_{x} k_{x} x}}{{K_{0} }}$	Mean predation/parasitism/nonreproductive host killing time
$k_{m} = \frac{{{\text{ln}}\left( {K_{0} } \right)}}{{T_{k} }}$	Pest kill rate (discretized assessment)

When individual daily predation and parasitism data are available, pest kill rates values can also be calculated with the Euler-Lotka Eq. ^58,59^, which is a more accurate method than the Birch approach^16^. In that case *r*_*m*_ can be assessed with Eq. (1) and *k*_*m*_ with Eq. (2). The values for *r*_*m*_ and *k*_*m*_ are obtained by iteration, i.e. by updating *r*_*m*_ and *k*_*m*_ values until the formula gives the required value of 1.

1 $$1 = \mathop \sum \limits_{x = 0}^{{x_{max} }} l_{x} m_{x} {\text{exp}}\left( { - r_{m} x} \right)$$

2 $$1 = \mathop \sum \limits_{x = 0}^{{x_{max} }} l_{x} k_{x} {\text{exp}}\left( { - k_{m} x} \right)$$

For all predators and parasitoids, the intrinsic rate of natural increase and the pest kill rate was calculated with both the Birch and Euler-Lotka approach as explained in van Lenteren et al.^16^. The *r*_*m*_ and *k*_*m*_ calculations are provided as supplementary material (supplementary material Tables S1–S14).

## Statistics

The Pearson’s product-moment correlation^60^ was used to calculate the correlations coefficients (PPMCC) between *r*_*m*_ values, *k*_*m*_ values and the *r*_*m*_ − *k*_*m*_ values.

## Supplementary Information

Supplementary Information 1.

Supplementary Information 2.

Supplementary Information 3.

Supplementary Information 4.

Supplementary Information 5.

Supplementary Information 6.

Supplementary Information 7.

Supplementary Information 8.

Supplementary Information 9.

Supplementary Information 10.

Supplementary Information 11.

Supplementary Information 12.

Supplementary Information 13.

Supplementary Information 14.

Supplementary Information S15, S16, S17.
