# Supplementary material for: The pest kill rate of thirteen natural enemies as aggregate evaluation criterion of their biological control potential of Tutaabsoluta
Source: Sci Rep. 2021 May 24;11:10756. doi: 10.1038/s41598-021-90034-8 (PMC8144571; doi:10.1038/s41598-021-90034-8)
Supplement: Supplementary file 15 — Supplementary Information S15, S16, S17. [file 41598_2021_90034_MOESM15_ESM.pdf]

Supplementary information

The online version contains supplementary material available at ***

Files S1-14 are Excel tables and are provided as files

supplementary material Table S1 kill rate *Bracon nigricans*
supplementary material Table S2 kill rate *Campyloneuropsis infumatus*
supplementary material Table S3 kill rate *Dineulophus phthorimaeae*
supplementary material Table S4 kill rate *Dolichogenidea gelechiidivoris*
supplementary material Table S5 kill rate *Engytatus varians*
supplementary material Table S6 kill rate for *basicornis*
supplementary material Table S7 kill rate *Macrolophus pygmaeus*
supplementary material Table S8 kill rate *Nesidiocoris tenuis*
supplementary material Table S9 kill rate *Necremnus tutae*
supplementary material Table S10 kill rate *Pseudapanteles dignus*
supplementary material Table S11 kill rate *Trichogramma pretiosum* without food
supplementary material Table S12 kill rate *Trichogramma pretiosum* with food
supplementary material Table S13 kill rate *Trichogrammatoidea bactrae*
supplementary material Table S14 kill rate *Tupiocoris cucurbitaceus*

Files S15-17 are presented below
supplementary material Text S15: S15 Supplementary text Differences in experimental conditions
supplementary material Table and Text S16: S16 Summary of data of intrinsic rate of population increase of *Tuta absoluta*
supplementary material Table S17: S17 Tomato pests and their natural enemies in Brazil

supplementary material **Text S15**

S15 Supplementary text Differences in experimental conditions

The lifetable data presented as supplementary material files S 1-14 and the values for r_m and k_m given in Tables 2 and 3 resulted from experiments under similar temperature conditions (24-26°C). The relative humidity conditions were not always similar and varied between 45 and 80%, but it is not expected that this variation is of great influence, though no scientific proof is available to support this assumption. However, development, predation and parasitism all take place on the plant-leaf surface and within enclosed experimental arenas with different levels of ventilation. It is supposed that under these conditions humidity on the leaf surface is higher than that of the ambient climate room environment. Also, photoperiods varied (range from 12-12 to 16-8 L:D) and light intensities were not similar. These differences may have influenced rates of predation and parasitism if the natural enemies are only active during the light period.

Nocturnal predation rates for the three Neotropical mirid predators (*C. infumatus*, *E. varians* and *M. basicornis*) were similar to values for predation rates obtained during the daylight period (Broekhuizen 2017). Therefore, it is supposed that the predation data for all six mirid species for which r_m and k_m values are presented in this paper are not influenced by different photoperiods during the experiments.

Nocturnal parasitism was not tested for any of the parasitoid species discussed in this paper. If parasitoids do not parasitize during the night, this may have a larger influence on daily rates of parasitism for proovigenic species than for synovigenic ones. Proovigenic parasitoids have all or a major part of their eggs matured when the females emerge and their daily number of eggs laid will be determined, among others, by the length of the day. The experiments with the proovigenic *D. gelechiidivoris* took place with a 12 h photoperiod, while those with *T. pretiosum* and *T. bactrae* had a 14 h period. As a result, the r_m and k_m values for the larval parasitoid *D. gelechiidivoris* might be slightly underestimated when compared with the two egg parasitoid species because the larval parasitoid had fewer hours available for parasitism and the total amount of eggs laid may, as a result, have been realized after more days than with a 14 h photoperiod. Synovigenic parasitoids usually need food during their adult life in order to be able to mature eggs and generally produce a limited number of eggs per day, depending on the rate of egg maturation. Thus, synovigenic parasitoids may still lay all their mature eggs at the shortest photoperiod to which they were exposed, i.e. in all but one case 14 hours, and it is supposed that their r_m and k_m values are not strongly influenced by the length of the photoperiod.

Reference: Broekhuizen, T.M. Prey selection, predation during night and oviposition site selection by three Neotropical mirids. MSc thesis, Wageningen University, The Netherlands, 49 pp. (2017).

supplementary material Table and Text S16

S16 Summary of data of intrinsic rate of population increase of *Tuta absoluta*

The published r_m values given in the table below vary between 0.074 and 0.19, with most values in the range of 0.13-0.19. The differences in r_m values may be the result of many causes, including different origin and rearing histories of *T. absoluta* populations, different tomato cultivars expressing varying degrees of host plant resistance, large differences in experimental procedures, and different ways of calculating the population parameters. The value provided for *T. absoluta* in Table 3 is one of the highest found. Most other r_m were determined by the Birch approach and values will be slightly higher when calculated with the Lotka-Euler approach. As no raw data for lifetables of the pest were available in the papers summarized in the table below, the Lotka-Euler approach could not be applied. In the discussion section of the paper an r_m of the pest in the range of 0.13-0.19 is used for comparison with the k_m of the natural enemies. However, when speculating about control capabilities of natural enemies, determination of the r_m of the pest on the tomato cultivars locally used is relevant.

Published r_m values of for *Tuta absoluta* (experiments at 24-26 ^{o}C)

**Cultivar**	R_0	T	r_m Birch	r_m Lotka-Euler	λ	**Reference**
Marmande	36.53	25.12	0.1432		1.15	Campos et al. 2020
Newton	55.8	23.7	0.169			Cekin and Yasar 2015
Caracas	47.5	24.3	0.159			Cekin and Yasar 2015
Torry	64.6	24.1	0.173			Cekin and Yasar 2015
Simsek	35.8	23.9	0.150			Cekin and Yasar 2015
?	42.01	28.25	0.132		1.141	Erdogan and Babaroglu 2014
Atabay	24.54	23.77	0.1336		1.1430	Gharekhani and Salek-Ebrahim 2014
Cluse	19.17	23.83	0.1222		1.1301	Gharekhani and Salek-Ebrahim 2014
Perenses	30.46	24.28	0.1389		1.1492	Gharekhani and Salek-Ebrahim 2014
NS-501	52.15	28.24		0.140	1.150	Kanle Satishchandra et al. 2019
Santa Clara			~ 0.11			Martins et al. 2016
Platense	48.92	27.98		0.14		Pereyra and Sánchez 2006
Sweakar-448	40.18	28.94	0.19		1.20	Rasheed et al. 2018
Falkato	15.73	30.09	0.09		1.095	Rostami et al. 2017
Isabella	17.66	30.22	0.095		1.099	Rostami et al. 2017
Grandella	10.03	31.12	0.074		1.076	Rostami et al. 2017
Tex 317	40.10	24.60	0.15		1.16	Silva et al. 2015
Bravo	83.07	23.80	0.1859	0.1908	1.2103	van Lenteren et al. 2019; Silva et al. 2015

?	92.25	31.96	0.132	1.14	Younes et al. 2019

R_0= net reproductive ratio; T= mean generation time; r_m= intrinsic rate of increase; λ= finite rate of increase

References

Campos, M.R. de, Béarez, P., Amiens-Desneux, E., Ponti, L., Gutierrez, A.P., Biondi, A., Adiga, A., Desneux, N. (2020). Thermal biology of *Tuta absoluta*: demographic parameters and facultative diapause. *Journal of Pest Science*, in press. https://doi.org/10.1007/s10340-020-01286-8

Cekin D., Yasar, B. (2015). The life table of *Tuta absoluta* (Meyrick, 1917)(Lepidoptera: Gelechiidae) on different tomato varieties. *Journal of Agricultural Sciences 21*, 199-206.

Erdogan, P., Babaroglu, N. E. (2014). Life table of the tomato leaf miner, *Tuta absoluta* (Meyrick) (Lepidoptera: Gelechiidae). *Journal of Agricultural Faculty of Gaziosmanpasa University, 31*, 80–89. https://doi.org/10.13002/jafag723

G. H. Gharekhani, G.H., Salek-Ebrahim, H. (2014). Life table parameters of *Tuta absoluta* (Lepidoptera: Gelechiidae) on different varieties of tomato. *Journal of Economic Entomology 107*, 1765-1770; DOI: http://dx.doi.org/10.1603/EC14059

Kanle Satishchandra, N., Chakravarthy, A.K., Özgökçe, M.S., Atlihan, R. (2019). Population growth potential of *Tuta absoluta* (Meyrick) (Lepidoptera: Gelechiidae) on tomato, potato, and eggplant. *Journal of Applied Entomology 2019*, 1–9. https://doi.org/10.1111/jen.12622

Martins, J.C., Picanco, M.C., Bacci, L., Guedes, R.N.C., Santana Jr, P.A., Ferreira, D.O., Chediak, M. (2016). Life table determination of thermal requirements of the tomato borer *Tuta absoluta*. *Journal of Pest Science 89*, 897–908. DOI 10.1007/s10340-016-0729-8

Pereyra, P. C., Sánchez, N. E. (2006). Effect of two solanaceous plants on developmental and population parameters of the tomato leaf miner *Tuta absoluta* (Meyrick) (Lepidoptera: Gelechiidae). *Neotropical Entomology, 35*, 671–676. https://doi.org/10.1590/s1519-566x2006000500016

Abdul Rasheed, V., Koteswara Rao, S.R., Ramesh Babu, T., Murali Krishna, T., Bhaskara Reddy, B.V., Mohan Naidu, G. (2018). Age specific fecundity life table of South American tomato leaf miner, *Tuta absoluta* (Meyrick) on tomato. *Journal of Entomology and Zoology Studies 6*, 546-549.

Rostami, E., Madadi, H., Abbasipour, H., Allahyari, H., Cuthbertson, A.G.S. (2017). Life table parameters of the tomato leaf miner *Tuta absoluta* (Lepidoptera: Gelechiidae) on different tomato cultivars. *Journal of Applied Entomology 141*, 88-96. DOI: 10.1111/jen.12319

Silva, D.B., Bueno, V.H.P., Lins Jr, J.C., van Lenteren, J.C. (2015). Life history data and population growth of *Tuta absoluta* at constant and alternating temperatures on two tomato lines. *Bulletin of Insectology 68*, 223–232.

van Lenteren, J.C., Bueno, V.H..P., Burgio, G., Lanzoni, A., Montes, F.C., Silva, D.B., de Jong, P.W., Hemerik, L. (2019). Pest kill rate as aggregate evaluation criterion to rank biological control agents: a case study with Neotropical predators of *Tuta absoluta* on tomato. *Bulletin of Entomological Research 109*, 812-820. doi:10.1017/S0007485319000130

Younes, A.A., Zohdy, N.Z.M., Abulfadl, H.A., Fathy, R. (2019). Life table parameters of the tomato leafminer, *Tuta absoluta* (Lepidoptera: Gelechiidae), on three solanaceous host plants. *African Entomology 27*, 461-467. doi.org/10.4001/003.027.0461

supplementary material Table S17

S17 Tomato pests and their natural enemies in Brazil

Pests occurring in tomatoes in Brazil and their natural enemies. Source for pests: Prattisoli and de Carvalho, 1995; source for natural enemies:V.H.P. Bueno and F.C. Montes, personal communication 2020.

Pest species	Candidate natural enemies when mirids are not available	Role of mirids
Bemisia tabaci	*Encarsia formosa, Eretmocerus* spp., *Amitus fuscipennis*	+++
Helicoverpa armigera	*Campoletis chloridae*, *Trichogramma* sp, Chrysopidae spp.	+++
Helicoverpa zea	*Trichogramma* spp., Chrysopidae spp.	?
Liriomyza spp.	Parasitoid spp.	?
Macrosiphum euphorbiae	Parasitoid spp.	+
Myzus persicae	Parasitoid spp.	+
Neoleucinodes elegantalis	Parasitoid spp.	+++
Phthorimaea operculella	*Trichogramma* spp., entomopathogenic virus	+++
Spodoptera frugiperda	*Trichogramma* spp., entomopathogenic virus	++
Tetranychus urticae	*Neoseiulus californicus* , *Phytoseiulus macropilis*	+
Trichoplusia ni	*Bacillus thuringiensis*, entomopathogenic virus	?
Tuta absoluta	*Trichogramma* spp., other parasitoid spp.	+++
Thrips spp.	*Orius insidiosus*	?

+++ = very good predation by mirids, ++=good predation by mirids, +=predation found but still needs to be quantified, ?=not yet tested
